# A Brief Web-Based Screening Questionnaire for Common Mental Disorders: Development and Validation

**DOI:** 10.2196/jmir.1134

**Published:** 2009-07-24

**Authors:** Tara Donker, Annemieke van Straten, Isaac Marks, Pim Cuijpers

**Affiliations:** ^2^Institute of PsychiatryKings College LondonLondonUK; ^1^Department of Clinical PsychologyVU University AmsterdamAmsterdamThe Netherlands

**Keywords:** Depression, anxiety, mental disorders, common, needs assessment, Internet, online systems

## Abstract

**Background:**

The advent of Internet-based self-help systems for common mental disorders has generated a need for quick ways to triage would-be users to systems appropriate for their disorders. This need can be met by using brief online screening questionnaires, which can also be quickly used to screen patients prior to consultation with a GP.

**Objective:**

To test and enhance the validity of the Web Screening Questionnaire (WSQ) to screen for: depressive disorder, alcohol abuse/dependence, GAD, PTSD, social phobia, panic disorder, agoraphobia, specific phobia, and OCD.

**Methods:**

A total of 502 subjects (aged 18 - 80) answered the WSQ and 9 other questionnaires on the Internet. Of these 502, 157 were assessed for DSM-IV-disorders by phone in a WHO Composite International Diagnostic Interview with a CIDI-trained interviewer.

**Results:**

Positive WSQ “diagnosis” had significantly (*P* < .001) higher means on the corresponding validating questionnaire than negative WSQ “diagnosis”. WSQ sensitivity was 0.72 - 1.00 and specificity was 0.44 - 0.77 after replacing three items (GAD, OCD, and panic) and adding one question for specific phobia. The Areas Under the Curve (AUCs) of the WSQ’s items with scaled responses were comparable to AUCs of longer questionnaires.

**Conclusions:**

The WSQ screens appropriately for common mental disorders. While the WSQ screens out negatives well, it also yields a high number of false positives.

## Introduction

The thriving development of Internet-based self-help aids [1] for particular mental disorders [2,3] has generated a need for quick ways to triage would-be users to systems appropriate for their disorders. Many sufferers do not easily recognize their particular mental problem [4] and could be guided by a Web-screening questionnaire to a self-help system appropriate for their problem. This could reduce the likelihood of their becoming disenchanted with using a self-help system not intended for their disorder. Such a questionnaire would preferably be conducted via the Internet, as it offers quick and easy access to large numbers of users at a low cost [5,6]. This kind of questionnaire could also assist professionals such as general practitioners (GPs) in screening their patients prior to consultation.

The screening must be brief, as subjects will undergo screening more readily if it is short, quick [7], and easy to read. A few brief online screening questionnaires [8-10] appear to be reliable and valid. The Internet-based Self-assessment Program for Depression (ISP-D [10]), for example, reported sensitivity, specificity, positive predictive values (PPV), and negative predictive values (NPV) for major depressive disorder of 0.82, 0.73, 0.67, and 0.86, respectively [10]. Sensitivity of another online test, the Web-Based Depression and Anxiety Test (WB-DAT [8]), ranged from 0.71 to 0.95, while specificity ranged from 0.87 to 0.97 for major depressive disorder (MDD), obsessive compulsive disorder (OCD), post-traumatic stress disorder (PTSD), panic disorder with and without agoraphobia, and social phobia. Sensitivity for generalized anxiety disorder (GAD) was somewhat lower (0.63). However, existing online screening questionnaires do not assess all mental disorders for which self-help systems are now being created. To reduce this paucity, we developed a brief online screening questionnaire which screens for different mental disorders: the Web Screening Questionnaire for common mental disorders (WSQ), based on the Screening Questionnaire (SQ) of Marks and colleagues [9]. The WSQ contains only 15 items and screens for depression, GAD, panic disorder with and without agoraphobia, social phobia, specific phobia, OCD, PTSD, and alcohol abuse/dependence. This paper reports optimization and validation of the WSQ.

## Methods

### Participants and Procedure

Participants were recruited (between May and December 2007) from the general Dutch population by using Internet banners (eg, Google and Dutch Internet sites on mental health issues). The advertisements linked to a Web page containing information about common mental disorders, Internet treatment and this study, an application form, and a link to the questionnaires. Subjects were asked to input their name and email address, so they could be identified and added to the data pool only once.

We specifically targeted adults (18 years of age or older) with Internet access and who felt anxious, depressed, or thought of themselves as drinking too much alcohol. We targeted a population with a high rate of common mental disorders as the kind likely to use the WSQ in the future. Since this population can only illuminate false negative and true positive rates, we needed controls to test those rates. Therefore, we also recruited 20 undergraduate psychology students who were not required to have symptoms, using banners at the VU University’s students’ Web page seeking participants for VU studies.

We excluded people reporting a high suicide risk (ie, a score of 3 on Q15 of the WSQ); they were advised to contact their GP. To raise the response rate, participants were told in advance that completers of the screening questionnaires would be offered a self-help book for common mental problems. Students received academic credit for participating. The study protocol was approved by the Medical Ethics Committee at the VU Medical Centre in Amsterdam, Netherlands.

**Figure 1:**
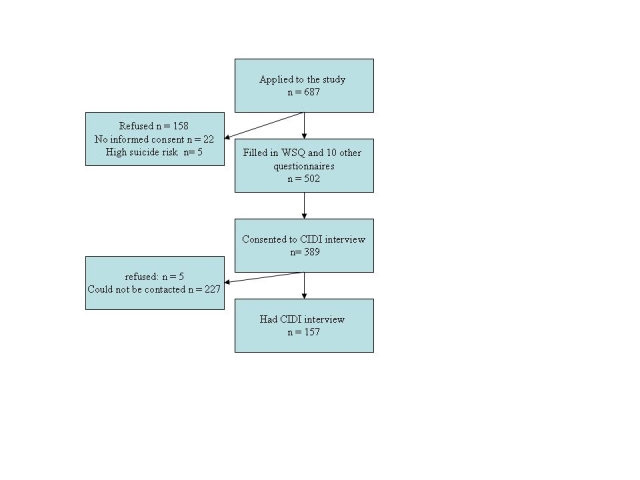
Flowchart of participants (WSQ)

Our study tested the WSQ’s validity and consisted of two parts (Figure 1):

1. Completion of 10 sets of questions: Internet demographic questions, the WSQ, and other questionnaires for common mental disorders: Center for Epidemiological Studies Depression scale (CES-D [11]), Generalized Anxiety Disorder scale (GAD-7 [12]), Fear Questionnaire (FQ [13]) plus a further question about eight kinds of specific phobia, Panic Disorder Severity Scale - Self Report (PDSS-SR [14,15]), Yale-Brown Obsessive Compulsive Scale (YBOCS [16,17]), Impact of Events Scale (IES [18]), Alcohol Use Disorders Identification Test (AUDIT [19]; details below),
2. A DSM-IV-diagnostic phone interview with a Composite International Diagnostic Interview (CIDI)-trained interviewer (CIDI lifetime, World Health Organization (WHO) version 2.1 [20]) to assess the presence of a current (ie, within the last 6 months) DSM-IV diagnosis [21] of MDD, dysthymia (Dyst), minor depression (MinD), social phobia, GAD, panic disorder, agoraphobia, specific phobia, OCD, PTSD, and alcohol abuse/dependence. CIDI-interviewers were blind to the subjects’ self-reports and the inclusion of control subjects (undergraduate psychology students).

In all, 687 people applied for the study, of whom 185 (27%) were excluded because they represented a high suicide risk (n = 5); there was no written informed consent (n = 22); or they refused to participate (n = 158). This left 502 participants, of whom 389 consented to a diagnostic phone interview, but 232 (60%) of those 389 either could not be contacted (n = 227) or refused (n = 5), leaving 157 participants who were phoned by a CIDI-trained interviewer within a mean of 13 days.

If participants had never experienced a traumatic event, they skipped the IES; if they had never drunk alcohol, they skipped the AUDIT; and if they had never suffered a panic attack, they skipped the PDSS-SR. Those who completed the screening questionnaires and gave informed consent entered the study.

### Measures

#### Development of the Web Screening Questionnaire for Common Mental Disorders (WSQ)

The WSQ for common mental disorders [22] has 15 self-rated questions based on the screening questionnaire (SQ) of Marks and colleagues [9] which screens for most common mental disorders. Of the SQ’s original questions we used 6 unchanged (WSQ Q1, 3, 5, 6, 11, and 15) and added 8 questions from further reliable and valid instruments. These are:

- WSQ Q2 for depression, from CIDI [20],
- WSQ Q4, 8, 9, 10, and 12 (for panic, social phobia, PTSD, and OCD from Mini-International Neuropsychiatric Interview (M.I.N.I. [23]), and
- WSQ Q13 and 14 (for alcohol, from AUDIT [19]).

Three questions of the original WSQ reached either low specificity or low sensitivity. To enhance validity, we used logistic regression analysis to determine whether other items from appropriate questionnaires could replace these WSQ-items. We amended three questions using items for GAD (WSQ Q3, from GAD-7 [12]) for panic (WSQ Q4 from PDSS-SR [15]), and for OCD (WSQ 12, from YBOCS [16,17]). We also added one question for the WSQ subscale specific phobia (WSQ Q7) which concerned further types of specific phobia. Each WSQ subscale has 1 - 2 items (for GAD, panic disorder, OCD, alcohol addiction, depression, agoraphobia, specific phobia, social phobia, and PTSD). Of the 15 WSQ questions, 8 had “yes” or “no” answers while the other 7 were Likert-type scales.

#### Further Screening Questionnaires

##### Depressive Symptoms

The Dutch version of the CES-D [11] has 20 self-rated items with each scored on a range of 0 - 3 and a total score of 0 - 60. The paper-pencil CES-D has good psychometric properties with a cut-off score of 16 [24]. The Internet CES-D is also reliable and valid with a cut-off score of 22 (Cronbach alpha: .93; sensitivity: 0.90; specificity: 0.74 [25]).

##### GAD

We translated the GAD-7 [12] into Dutch for self-rating of generalized anxiety symptoms. Each of its 7 questions is rated 0 - 3 (“not at all” to “nearly every day”), and the total score range is 0 - 21. Reliability is excellent (Cronbach alpha = .92). With a cut-off point of ≥ 10, sensitivity is 0.89 and specificity is 0.82 among primary care participants [12]. The GAD-7 was translated into Dutch by forward-translation (translated and discussed by two independent health professionals) and blind backward-translation (by an independent translator whose mother tongue is English). Since psychometric properties may differ among other populations, the Dutch version of the GAD-7 is validated in another study (TD, AVS, IMM, and PC, unpublished data, 2009).

##### Panic Disorder

The Dutch version of the PDSS [14] self-report SR form [15] asks 7 questions about 7 dimensions of panic disorder, each self-rated 0 - 4, with a total score range of 0 - 28. With a cut-off score of 8, sensitivity is 0.83 and specificity is 0.64 [14].

##### Phobias (Agoraphobia, Social Phobia, Specific Phobia)

The Dutch version of the FQ [13] detects agoraphobia, social phobia, and blood-injury phobia. The FQ’s total phobia scale contains 15 items; each self-rated 0 - 8, with a total score range of 0 - 120. Several studies support the validity of the FQ’s social and agoraphobia subscales [26-29]. To the FQ’s 5 blood/injury questions we added a single self-rated question (“are you scared of …?”) concerning further types of specific phobias, to be ticked as present or absent: animals (eg, dogs, cats), natural events (eg, earthquakes, storms, flooding), body fluids (eg, faeces, vomit, semen), materials (eg, cleaning products, medicine, poison), medical appointments (eg, dentist, hospital), items at home (eg, telephone, toilet, soap), specific situations (eg, driving, riding elevators, crossing bridges), and other (eg, vomiting, children). We omitted the FQ’s 6 anxiety-depression items.

##### PTSD

The IES [18] assesses signs and symptoms of avoidance and intrusion after a serious or traumatic life event. It has 15 items, each self-rated 0 - 5, with a total score range of 0 - 75. People who score ≥ 26 are likely to have PTSD. The Dutch version is reliable and sensitive [30].

##### OCD

We used the Dutch 10-item severity subscale of the YBOCS [16,17]. Each self-rated item is rated 0 - 4, with a total score range of 0 - 40. Tests of internal consistency of the total scale (Dutch version) are .69 to .91 (Cronbach alpha) and compare well with several but not all measures often used to assess OCD [31]. A total score of 13 or more denotes clinically significant obsessive-compulsive symptoms [32].

##### Alcohol Abuse/Dependence

The Dutch version of WHO’s self-rated AUDIT [19] identifies people with hazardous alcohol consumption and dependence in primary care. Each of its 10 items is rated 0 - 4, with a total score range of 0 - 40. Cronbach alpha is .65 to .93: overall sensitivity is 0.92, and specificity is 0.94 [19]. A cut-off score of 8 is recommended for various endpoints (eg, alcohol-related social problems or medical problems) [33].

##### Diagnoses

We used the Lifetime version 2.1 of the CIDI [19] in its Dutch version [34,35] as a “gold standard’ to assess the presence of DSM-IV disorders in the last 6 months (GAD, panic disorder, OCD, alcohol abuse/dependence, MDD, Dyst, MinD, agoraphobia, specific phobia, social phobia, and PTSD). The CIDI is reliable and valid [36,37]. The CIDI was administered by phone by trained CIDI interviewers who were psychologists or master’s-level psychology students. The CIDI interviews used in this trial lasted 69 minutes on average.

### Analyses

To establish whether WSQ scores differed significantly between subjects with positive and with negative screen results, we conducted t-tests on the mean and standard deviation of each screening instrument separately. In the sub-sample that had a diagnostic interview, we performed chi-square tests to ascertain whether WSQ scores differed between subjects with and without DSM-IV disorders.

We calculated sensitivity and specificity, and positive and negative predictive values, for each WSQ subscale regarding its corresponding DSM-IV disorder (predictive validity). Sensitivity is the probability that a person who has a disorder is screen positive. Specificity is the probability that a person not suffering from a disorder is screen negative. There is no consensus of what levels of sensitivity and specificity are acceptable, as they depend on the test’s aim, costs, and benefits [38]. The WSQ aims to detect clinically-relevant mood, anxiety, and alcohol-related problems. Therefore, to minimize missed cases we set threshold levels of sensitivity at 0.70 or more, and of specificity at 0.40 or more. PPV is the probability of a positive diagnosis after a positive screening, and NPV is the probability of a negative diagnosis after a negative screening. PPV and NPV depend on prevalence (PPV increases when prevalence increases), so we did not set acceptable levels of these.

For WSQ questions which turned out to have unacceptable sensitivity or specificity, we replaced them with relevant items from the appropriate screening questionnaire. To find which items best predicted the chance of detecting a diagnosis, we used logistic regression analyses (Forward Likelihood Ratio method). We replaced items only if they improved validity. We calculated the Area Under the Curve (AUC) for the WSQ’s scaled and dichotomous response options and its appropriate screening questionnaires. The AUC (the sum of sensitivity versus [1 – ] specificity) measures a scale’s accuracy; it equals the probability that a randomly chosen case will score higher than a randomly chosen non-case [39]. AUCs of 0.5 - 0.7 are said to reflect low accuracy, 0.7 - 0.9 moderate accuracy, and 0.9 - 1.0 high accuracy [40]. Furthermore, we performed t-tests and χ² tests to examine differences in demographic and questionnaire results between subjects who had a CIDI diagnostic interview and those who did not, and tests to examine whether a student sub-sample’s WSQ scores differed from the whole sample.

Our analyses used diagnoses reached within the last 6 months. MDD, Dyst, and MinD were combined into the category depressive disorder. For all analyses we used SPSS version 15.0 for Windows.

## Results

### Sample Characteristics

The total sample (N = 502) had a mean age of 43 years (SD 13, range 18 - 80), and 285 (57%) of the subjects were female. Of the 157 subjects who had a CIDI interview, the mean age was 43 (SD 15, range 18 - 80). Of these, 89 (57%) were female, and 107 (68%) subjects met DSM-IV criteria for any current (ie, within the past 6 months) depressive disorder, anxiety disorder, and/or alcohol abuse/dependence. A total of 67 (43%) subjects had more than one diagnosis (Table 1).

**Table 1:** Demographic characteristics and prevalence of diagnosis

		N (%)	
		Complete sample	CIDI sub-sample
Completed all questionnaires on Internet		502 (100)	157
**Gender, N (%)**			
	Male	217 (43)	68 (43)
	Female	285 (57)	89 (57)
**Age, Mean (SD)**		43 (13)	43 (15)
	(Range)	(18 - 80)	(18 - 80)
**Education**			
	Low^a^	99 (20)	27 (17)
	Medium^b^	217 (43)	73 (47)
	High^c^	186 (37)	57 (36)
**Country**			
	Netherlands	474 (94)	146 (94)
	Other	28 (6)	11 (6)
**Marital status**			
	Single	180 (36)	65 (41)
	Married or cohabiting	241 (48)	67 (43)
	Divorced/widowed	81 (16)	25 (16)
DSM-IV diagnosis within last 6 months, on CIDI phone interview		157	
Any depressive disorder		52 (33)	
	Major depressive disorder	46 (29)	
	Dysthymia	9 (6)	
	Minor depression	8 (5)	
Any anxiety disorder		94 (60)	
	Social phobia	32 (20)	
	GAD	30 (19)	
	Panic disorder	10 (6)	
	Panic with agoraphobia	22 (14)	
	Agoraphobia	10 (6)	
	Specific phobia	40 (26)	
	Obsessive-compulsive disorder	10 (6)	
	PTSD	12 (8)	
Alcohol abuse/dependence		23 (15)	
Any disorder		107 (68)	
> one diagnosis		67 (43)	

^a^Low education: primary and lower general secondary education.

^b^Medium education: Intermediate Vocational Training,school of higher general secondary education or pre-university education.

^c^High education: higher vocational education or university.

### Comparisons of WSQ With Other Questionnaires

Table 2 shows that subjects who scored “Yes” for any particular WSQ “diagnosis” had significantly higher means (*P* < .001) on the corresponding validating questionnaire than those who scored “No” for that WSQ “diagnosis”.

**Table 2:** WSQ and screening questionnaires: means, standard deviations (SDs) and prevalence (N = 502)

Other screening questionnaires:	“Diagnosis” on WSQ (Web Screening Questionnaire)					
	Yes		No			t (d.f. = 500)
	N(%)	M (SD)		N (%)	M (SD)	
**Any depressive disorder**					
CES-D (score range 0 - 60)	296 (59.0)	32.2 (7.1)		206 (41.0)	18.1 (10.3)	15.2^a^
**Generalized anxiety disorder**					
GAD-7 (score range 0 - 21)	320 (63.8)	13.6 (3.9)		182 (36.3)	5.5 (3.1)	24.3 ^a^
**Panic disorder (without agoraphobia)**					
PDSS-SR (score range 0 - 28)	278 (55.4)	9.3 (5.1)		224 (44.6)	0.6 (1.7)	24.2 ^a^
**Panic with agoraphobia**					
PDSS-SR	153 (30.5)	11.2 (5.1)		349 (69.5)	2.9 (4.1)	19.3 ^a^
**Agoraphobia (without panic disorder)**					
FQ-agoraphobia (score range 0 - 40)	205 (40.8)	12.7 (10.9)		297 (59.2)	2.9 (4.5)	14.0 ^a^
**Social phobia**					
FQ-social phobia (score range 0 - 40)	226 (45.0)	16.6 (8.7)		276 (55.0)	7.0 (6.0)	14.6^a^
**Specific phobia**					
FQ-specific phobia^b^ (score range 0 - 40)	290 (57.8)	7.6 (7.7)		212 (42.2)	2.3 (3.5)	9.2 ^a^
**Obsessive-compulsive disorder**					
YBOCS (score range 0 - 40)	182 (36.3)	11.0 (6.3)		320 (63.8)	0.8 (2.3)	26.2 ^a^
**Post-traumatic stress disorder**					
IES (score range 0 - 75)	273 (54.4)	33.5 (20.1)		229 (45.6)	0.0^c^	25.3 ^a^
**Alcohol abuse/dependence**					
AUDIT (score range 0 - 40)	198 (39.4)	19.6 (6.2)		260 (60.6)	6.3 (5.5)	24.4 ^a^

^a^Significant at *P* < .001.

^b^Additional specific phobia questions were dichotomous, so their means and standard deviations could not be calculated.

^c^If participants had never experienced a traumatic event then they skipped the IES.

### Predictive Validity and Refinement of the WSQ

For the three WSQ subscales, GAD, OCD, and panic, validity was below threshold levels of 0.70 for sensitivity and 0.40 for specificity, so we replaced those (based on logistic regression analysis) with relevant items from the appropriate screening questionnaires (GAD-7, YBOCS, and PDSS-SR, respectively). This improved sensitivity or specificity. The WSQ subscale-specific phobia had an unacceptably low sensitivity (0.60), but we did not replace it with an item from the appropriate screening questionnaire as that did not improve sensitivity or specificity.

Based on the log-likelihood ratio statistic, using logistic regression analyses, we added three categories of the specific phobia question, “Are you scared of …?”. These categories were (1) animals, (2) specific situations, and (3) medical issues, which improved the sensitivity of the WSQ subscale for specific phobia but not for specificity (sensitivity: from 0.60 to 0.80; specificity: from 0.77 to 0.47).

Table 3 shows that for all 10 CIDI DSM-IV diagnoses more subjects with a CIDI diagnosis scored positive on the corresponding WSQ questions than did subjects without that CIDI diagnosis. The differences were all significant at the *P* < .001 level except for specific phobia (*P* = .003). Table 3 also shows that the WSQ’s sensitivity ranged from 0.72 (social phobia) to 1.00 (agoraphobia). The WSQ’s specificity ranged from 0.44 (panic disorder) to 0.77 (panic disorder with agoraphobia). PPV varied from 0.11 (PTSD) to 0.51 (any depressive disorder), and NPV varied from 0.87 (specific phobia) to 1.00 (agoraphobia).

**Table 3:** WSQ vs CIDI diagnoses: prevalence, sensitivity, specificity, positive predictive value (PPV), and negative predictive value (NPV) (n = 157)

WSQ “Diagnosis”	CIDI DSM-IV Diagnosis							
		No	Yes	χ² (d.f. = 1)	Sensitivity	Specificity	PPV	NPV
		n	n					
**Any depressive disorder**							
WSQ-depression	No	62	8	26.8^a^	0.85	0.59	0.51	0.89
	Yes	43	44					
**Generalized anxiety disorder**							
WSQ-GAD	No	57	2	^b^	0.93	0.45	0.29	0.97
	Yes	70	28					
**Social phobia**							
WSQ-social phobia	No	91	9	22.0^a^	0.72	0.73	0.40	0.91
	Yes	34	23					
**Panic disorder**							
WSQ-panic	No	65	1	^b^	0.90	0.44	0.10	0.98
	Yes	82	9					
**Panic with agoraphobia**							
WSQ-panic+agoraphobia	No	104	3	^b^	0.86	0.77	0.38	0.97
	Yes	31	19					
**Agoraphobia**							
WSQ-agoraphobia	No	92	0	^b^	1.00	0.63	0.15	1.00
	Yes	55	10					
**Specific phobia**							
WSQ-specific phobia	No	55	8	9.1^c^	0.80	0.47	0.34	0.87
	Yes	62	32					
**Obsessive-compulsive disorder**							
WSQ-OCD	No	102	2	^b^	0.80	0.69	0.15	0.98
	Yes	45	8					
**Post-traumatic stress disorder**							
WSQ-PTSD	No	68	2	^b^	0.83	0.47	0.11	0.99
	Yes	77	10					
**Alcohol abuse/dependence**							
WSQ-alcohol	No	97	4	^b^	0.83	0.72	0.34	0.96
	Yes	37	19					

^a^Significant at *P* < .001.

^b^Not able to calculate χ² due to small numbers (< 5) in cells.

^c^Significant at *P* = .003.

Compared to the corresponding CIDI DSM-IV diagnoses, the AUC for the WSQ subscales with scaled responses (WSQ subscales GAD, OCD, alcohol, and panic) were similar to the AUC of the longer questionnaires, ranging from an AUC of 0.76 for the WSQ subscale panic versus an AUC of 0.70 of the PDSS-SR, to an AUC of 0.81 for the WSQ subscale OCD versus an AUC of 0.85 for the YBOCS. The AUC for the dichotomous WSQ’s subscales of panic with agoraphobia and agoraphobia were similar to the AUC of the longer, scaled questionnaires (PDSS: AUC of 0.79 versus WSQ panic with agoraphobia: AUC of 0.82; both WSQ and FQ subscale agoraphobia: AUC of 0.81), but not for the WSQ dichotomous subscales of depression, social phobia, and PTSD (ranging from WSQ subscale depression: AUC of 0.72 versus CES-D: AUC of 0.84 to WSQ subscale PTSD: AUC of 0.65 versus IES: AUC of 0.82) (Table 4).

**Table 4:** WSQ and screening questionnaires versus CIDI diagnoses: Area Under the Curve (AUC) and 95% CI for scaled and dichotomous response options

WSQ “Diagnosis”	CIDI DSM-IV Diagnosis	
	AUC	95% C.I.
**Any depressive disorder**	
WSQ-depression	0.72	0.64 - 0.80
CES-D	0.84	0.77 - 0.90
**Generalized anxiety disorder**	
WSQ-GAD	0.78	0.69 - 0.86
GAD-7	0.77	0.68 - 0.85
**Social phobia**	
WSQ-social phobia	0.72	0.62 - 0.82
FQ-social phobia	0.82	0.74 - 0.89
**Panic disorder**	
WSQ-panic	0.76	0.59 - 0.93
PDSS-SR	0.70	0.57 - 0.88
**Panic with agoraphobia**	
WSQ-panic+agoraphobia	0.82	0.72 - 0.91
PDSS-SR	0.79	0.69 - 0.89
**Agoraphobia**	
WSQ-agoraphobia	0.81	0.73 - 0.90
FQ-agoraphobia	0.81	0.70 - 0.91
**Obsessive-compulsive disorder**	
WSQ-OCD	0.81	0.65 - 0.97
YBOCS	0.86	0.72 - 0.99
**Post-traumatic stress disorder**	
WSQ-PTSD	0.65	0.51 - 0.80
IES	0.82	0.67 - 0.97
**Alcohol abuse/dependence**	
WSQ-alcohol	0.77	0.68 - 0.86
AUDIT	0.75	0.66 - 0.84

### Differences Between Students and Non-students

As expected, students compared to non-students had significantly lower scores on the WSQ subscales for depression (P = .004), alcohol (P < .001), GAD (P < .001), OCD (P < .001), panic (P < .001), and panic with agoraphobia (P = .004).

### Differences Between CIDI Interviewed and Non-interviewed Sub-samples

Demographic variables did not differ significantly between subjects who had a CIDI diagnostic interview and those who did not. However, those who had a CIDI interview scored significantly lower on one WSQ subscale (social phobia; P = .009), on the CES-D (P = .05), and on the FQ social-phobia subscale (P = .03).

## Discussion

### Principal Results

It takes about two minutes to complete the WSQ to detect common mental disorders. The WSQ quickly detects clinically-relevant mood, anxiety, and alcohol-related problems and so can guide Internet users to Internet-self-help modules appropriate for their problem, or quickly screen patients prior to consultation with a GP. This measure can also be used in more homogeneous samples to screen out people with co-morbid disorders. The WSQ turned out to be a valid screener for social phobia, panic disorder with agoraphobia, agoraphobia, OCD, and alcohol abuse/dependence (sensitivity: 0.72 - 1.00; specificity: 0.63 - 0.80), and appropriate for depressive disorder, GAD, PTSD, specific phobia, and panic disorder (without agoraphobia) (sensitivity: 0.80 - 0.93; specificity: 0.44 - 0.51) in our study population. Interestingly, the AUC’s of the WSQ’s scaled single items, and some of the dichotomous items, were comparable to the AUC’s of the longer questionnaires, supporting our conclusion that short questionnaires, sometimes with just one item, can be as valid as longer ones. This is in line with previous studies [7,41-43].

Compared to psychometric properties of other online screening questionnaires [8,10] (sensitivity: 0.63 - 0.95; specificity: 0.73 - 0.97), WSQ’s sensitivity was similar (sensitivity: 0.72 - 1.00), but specificity was, for some disorders, considerably lower (specificity: 0.44 - 0.80). One explanation for this lower specificity might be that we have used 6-month prevalence rates rather than point prevalence rates, whereas the WSQ assesses current symptoms rather than symptoms during the previous 6 months. Therefore, specificity might be higher when the WSQ is validated against concurrent DSM-IV diagnoses. Although only one of the two symptoms is required for a diagnosis of MDD, the “WSQ depression diagnosis” is based on elevated mood and anhedonia. However, when only one of the two symptoms would give a positive “WSQ depression diagnosis”, specificity was below the threshold level of 0.40. Therefore, both core depression symptoms are needed to fulfill the criteria of a positive “WSQ depression diagnosis”. Although sensitivity, specificity, and NPV’s were acceptable for most WSQ “diagnosis”, PPV’s were low (0.10 - 0.51), indicating that the WSQ misidentified many participants as (falsely) positive. NPV and PPV depend on prevalence. When prevalence is high, which might be the case in self-selected samples such as those in this study, “true” negatives will have a greater impact, and when prevalence is low, “true positives” have a higher impact on the NPV and PPV. When prevalence is low, a positive diagnosis from the WSQ should be regarded with caution. Subjects with a positive WSQ score can then undergo more in-depth screening with a longer questionnaire or CIDI with a higher specificity. However, the test successfully identified “true” negatives (high NPV), which is to say that subjects with no WSQ positive score (“diagnosis”) of any kind are likely to have no relevant DSM-IV diagnosis when interviewed by CIDI. In brief, the WSQ screens out negatives well but yields many false positives.

Although WSQ’s false positives do not have a diagnosis, they might have symptoms of depression, anxiety, or alcohol problems, since they have elevated scores on the relevant screening questionnaires.

### Limitations

One limitation of our study is that the CIDI-diagnosis live phone interviews were not taped, so inter-rater reliability could not be calculated. Second, subjects always completed the WSQ on the Internet before the other screening questionnaires, so order effects could not be ruled out. Third, though sensitivity and specificity do not depend on prevalence of the disorders in the population, the PPV and NPV do; consequently, the values we found might not generalize to situations where prevalence is different. Fourth, it is not known how representative our self-recruited participants are of Internet self-help applicants. Fifth, subjects who had a CIDI interview had significantly less social phobia on that WSQ-subscale than those who did not, so the WSQ-social-phobia results might be less generalizable to other populations. Sixth, as described earlier, 6-month prevalence rates of DSM-IV diagnoses were used, whereas the WSQ assesses current symptoms. Ideally, the WSQ should be validated against concurrent DSM-IV diagnoses. Seventh, norms are unavailable for acceptable levels of sensitivity and specificity which depend on the test’s aim, costs, and benefits [38]. As the WSQ aims to detect clinically-relevant mood, anxiety, and alcohol-related problems in order to minimize missed cases, we chose thresholds of sensitivity at 0.70 or more and of specificity at 0.40 or more. Finally, the WSQ for common mental disorders could be further simplified [44]. However, before using this simplified WSQ, psychometric properties have to be evaluated.

Despite its limitations, the WSQ is a useful and quick Internet screening tool to detect people likely to have common mental disorders.

### Future Research

Many false positives were found for WSQ subscales GAD, panic, specific phobia, and PTSD, while far fewer false positives were found for alcohol abuse/dependence, social phobia, panic disorder with agoraphobia, and OCD. The high rate of false positives may, for some questions, be due to a lack of clarity or classification criteria. Future research which enhances clarity of questions and classification criteria is needed to improve the predictive power of the WSQ.

## Multimedia Appendix 1

WSQ

## Abbreviations

AUC: area under the curve
AUDIT: alcohol use disorders identification test
CES-D: Center for Epidemiological Studies Depression scaleCIDI:composite international diagnostic interview
Dyst: dysthymia
DSM-IV: Diagnostic Statistical Manual, 4thedition
FQ: fear questionnaire
GAD: generalized anxiety disorder
GAD-7: generalized anxiety disorder - 7
GPs: general practitioners
IES: impact of events scale
ISP-D: Internet-based self-assessment program for depression
M: mean
MinD: minor depression
MDD: major depressive disorder
MINI: mini-international neuropsychiatric interview
NPV: negative predictive value
OCD: obsessive compulsive disorder
PDSS-SR: panic disorder severity scale self-report
PPV: positive predictive value
PTSD: Post-Traumatic Stress Disorder
SD: standard deviation
SQ: screening questionnaire
VU: Vrije Universiteit
WB-DAT: Web-based depression and anxiety test
WHO: World Health Organization
WSQ: Web screening questionnaire
YBOCS: Yale-Brown Obsessive Compulsive Scale
