# Supplementary material for: A Brief Web-Based Screening Questionnaire for Common Mental Disorders: Development and Validation
Source: J Med Internet Res. 2009 Jul 24;11(3):e19. doi: 10.2196/jmir.1134 (PMC2763401; doi:10.2196/jmir.1134)
Supplement: Supplementary file 1 [file jmir_v11i3e19_app1.pdf]

Multimedia Appendix 1. Web Screening Questionnaire for Common Mental Disorders (WSQ)

Q	Web Screening Questionnaire for common mental disorders (WSQ)	Sub-scale	From
1	Circle a number from the scale below to show how much you are troubled by feeling miserable or depressed:	Depres.	SQ
	Hardly at all (0) ☐ ; (1) ☐ ; Slightly disturbing/ not really disabling (2) ☐ ; (3) ☐ ; Definitely disturbing/ disabling (4) ☐ ; (5) ☐ ; Markedly disturbing/ disabling (6) ☐ ; (7) ☐ ; Very severely disturbing/ disabling (8) ☐		
2	Do you experience a loss of interest and/or pleasure in most things, like work, hobbies and other things you usually enjoy? Yes (1) ☐ No (0) ☐	Depres.	CIDI
3	During the past two weeks, how often have you been bothered by the following problem: Having trouble relaxing?	GAD	GAD-7
	Not at all (0) ☐ ; Several days (1) ☐ ; More than half the days (2) ☐ ; Nearly every day (3) ☐		
4	A panic attack is a sudden rush of fear or discomfort accompanied by at <u>least 4 of the symptoms</u> listed below. In order to qualify as a <u>sudden rush</u>, the symptoms must <u>peak within 10 minutes</u>.<br>Symptoms are: rapid or pounding heartbeat, sweating, trembling/shaking, breathlessness, feeling of choking, chest pain/discomfort, nausea, dizziness/faintness, feelings or unreality, numbness/tingling, chills or hot flashes, fear of losing control or going crazy, fear of dying.<br>If you have had any panic attacks during the past week, how distressing (uncomfortable, frightening) were they <u>while they were</u> happening? If you did not have any panic attacks but did have limited symptoms attacks, answer for the limited symptom attacks.	Panic	PDSS-SR
	Not at all distressing, or no panic or limited symptom attacks during the past week (0) ☐ ; Mildly distressing (not too intense) (1) ☐ ; Moderately distressing (intense, but still manageable) (2) ☐ ; Severely distressing (very intense) (3) ☐ ; Extremely distressing (extreme distress during all attacks) (4) ☐		
5	Do you avoid public places from which a quick escape may be difficult or do you endure this with clear suffering or anxiety? (e.g. public transport, shops/town centers, queues, cinema, unfamiliar buildings, distance from home). Yes (1) ☐ No (0) ☐	AGO	SQ
6	Are you either extremely anxious or do you avoid specific objects or situations? Yes (1) ☐ No (0) ☐	Specific Phobia	SQ
7	Are you scared of: <u>animals</u> (e.g. dogs, spiders, snakes, cats, birds, mice, insects) or <u>medical issues</u> (e.g. blood, dentist, injection, surgery, hospital, doctor) or <u>specific situations</u> (e.g. bus, crowded shop, tunnel elevator, airplane, bridge or car driving) Yes (1) ☐ No (0) ☐	Specific Phobia	VU
8	Have you avoided social situations for fear that attention might be on you? Yes (1) ☐ No (0) ☐	Social Phobia	MINI
9	Are you fearful or embarrassed being watched, being the focus of attention, or fearful of being humiliated? (This includes situations like speaking in public, eating in public with others, writing while someone watches, or being in social situations). Yes (1) ☐ No (0) ☐	Social Phobia	MINI
10	Did your symptoms start after having experienced, witnessed or had to deal with an extremely traumatic event that included actual or threatened death or serious injury to you or someone else? (e.g. serious accident, sexual or physical assault, a terrorist attack, being held hostage, kidnapping, hold-up, fire, discovering a body, unexpected death, war, natural disaster…) Yes (1) ☐ No (0) ☐	PTSD	MINI
11	Have you ever experienced a traumatic event? Yes (1) ☐ No (0) ☐	PTSD	SQ
12	Obsessions are recurrent thoughts, impulses or images that are unwanted, distasteful, inappropriate, intrusive or distressing (e.g. the idea of hurting your children although you know you never want to do that). How much time did you spend on obsessions in the past week?	OCD	ybocs
	0 hr/day or no obsessions (0) ☐ ; 0-1 hr/day (1) ☐ ; 1-3 hr/day (2) ☐ ; 3-8 hr/day ☐ (3) ; >8 hr/day ☐ (4)		
13	How many drinks containing alcohol do you have on a typical day when you are drinking?	Alcohol	audit
	None (0) ☐ ; 1-2 (1) ☐ ; 3-4 (2) ☐ ; 5-6 (3) ☐ ; 7-9 (4) ☐ ; 10 or more (5) ☐		
14	How often do you have six or more drinks on one occasion?	Alcohol	audit
	Never (0) ☐ ; Less than monthly (1) ☐ ; Monthly (2) ☐ ; Weekly (3) ☐ ; Daily or nearly daily (4) ☐		
15	Has the idea of harming yourself or taking your own life, recently come into your mind?	Suicide	SQ
	Definitely not (0) ☐ ; Has crossed my mind but I would not do it (1) ☐ ; I seriously considered it but I stopped myself (2) ☐ ; I would do it given the opportunity (3) ☐		

*WSQ cut-off scores: Depression: Q1≥ 5 & Q2=1; GAD: Q3≥2; Panic: Q4 ≥1; Panic with Ago Q4 ≥1 & Q5=1; Ago: Q5=1; Specific phobia: Q6 or Q7≥1; Social phobia: Q8=1 & Q9=1; PTSD: Q10=1 or Q11=1; OCD: Q12≥1; Alcohol Abuse/Dependence : Q13≥2 & Q14≥3 ; Suicide : Q15≥3 (exclusion)
